# Bioactive Lipid Signaling in Cardiovascular Disease, Development, and Regeneration

**DOI:** 10.3390/cells9061391

**Published:** 2020-06-03

**Authors:** Aaron H. Wasserman, Manigandan Venkatesan, Aitor Aguirre

**Affiliations:** 1 Regenerative Biology and Cell Reprogramming Laboratory, Institute for Quantitative Health Science and Engineering (IQ), Michigan State University, East Lansing, MI 48824, USA; awasserm@msu.edu (A.H.W.); venkat40@msu.edu (M.V.); 2 Department of Biomedical Engineering, College of Engineering, Michigan State University, East Lansing, MI 48824, USA

**Keywords:** bioactive lipid, heart, development, regeneration, stem cell, cardiovascular, cardiac regeneration

## Abstract

Cardiovascular disease (CVD) remains a leading cause of death globally. Understanding and characterizing the biochemical context of the cardiovascular system in health and disease is a necessary preliminary step for developing novel therapeutic strategies aimed at restoring cardiovascular function. Bioactive lipids are a class of dietary-dependent, chemically heterogeneous lipids with potent biological signaling functions. They have been intensively studied for their roles in immunity, inflammation, and reproduction, among others. Recent advances in liquid chromatography-mass spectrometry techniques have revealed a staggering number of novel bioactive lipids, most of them unknown or very poorly characterized in a biological context. Some of these new bioactive lipids play important roles in cardiovascular biology, including development, inflammation, regeneration, stem cell differentiation, and regulation of cell proliferation. Identifying the lipid signaling pathways underlying these effects and uncovering their novel biological functions could pave the way for new therapeutic strategies aimed at CVD and cardiovascular regeneration.

## 1. Introduction

Cardiovascular diseases (CVDs) are the leading cause of morbidity and mortality in the developed world. By 2035, in the United States alone, CVD economic burden is expected to increase to over $1 trillion dollars annually [1]. CVD appears in many forms, from congenital heart disease (CHD) in newborns to coronary artery disease, myocardial infarction (MI), heart failure, and hypertension in adults [1,2]. Treatment for these diseases remains challenging, and, in most cases, only symptomatic options are available. Therefore, effective approaches for the prevention and treatment of CVD are crucial to reduce the future disease burden and improve population health. Bioactive lipids are a chemically heterogeneous group of lipids with crucial signaling functions in body homeostasis and disease and are present in every organ. In classic biology, the predominant roles ascribed to lipids are three: (1) synthesis of cell membrane components (e.g., cholesterol, phospholipids); (2) energy production for cellular metabolism (e.g., fatty acids, triglycerides); (3) paracrine and autocrine cellular communication through bioactive lipids (e.g., sphingolipids, eicosanoids, steroids) [3]. The rapid emergence of lipidomics, due in no small part to advances in LC-MS and computational biology over the last two decades, has led to the rapid realization that hosts of new bioactive lipids with unknown function exist [4,5,6]. In many cases, these novel bioactive lipids are found in contexts that have not been observed traditionally (e.g., regeneration, stem cell fate), suggesting our knowledge of bioactive lipid biology is very limited [7].

Diet and nutrition play a significant role in bioactive lipid biology as mammals lack the enzymatic repertoire to synthesize the precursors for these messenger molecules endogenously ([8]; see further below for more detail on biosynthetic pathways). Bioactive lipids act predominantly via G-protein coupled receptors (GPCRs, e.g., the prostaglandin E receptor (*PTGER1 gene*, EP_1_ receptor)) or nuclear receptor (NR) binding (e.g., *PPARG*) [9,10], but other signal transduction mechanisms have been described, such as the regulation of ion channel activity [11]. Activating multiple signaling pathways and regulatory molecules downstream of their receptors can result in the regulation of a wide range of downstream signaling pathways [9], including GPCRs of the Gi, Gq, and G12/13 forms, phosphoinositide 3-kinase (PI3K)-to-Akt/protein kinase B (PKB), Ras/Raf-MEK-ERK, phospholipase C (PLC) to protein kinase C (PKC), Wnt/glycogen synthase kinase-3β (GSK-3β), Janus kinase (Jak)/signal transducer and activator of transcription 3 (Stat3), nuclear factor-κB (NF-κB), and interleukin-4 (IL-4) signaling [12,13,14,15]. In the cardiovascular system, bioactive lipids are involved in many diverse functions, including cardiovascular development [9,16], cardiac regeneration [17], inflammation [18], blood coagulation [19], blood vessel permeability [20], angiogenesis [21], control of vascular tone [22], and cellular migration and adhesion [23]. They also play a critical role in the diverse physiological functions and pathological conditions of various other human diseases, such as obesity [24], inflammation [18], diabetes [25], and cancer [26,27]. Several major groups of bioactive lipids, such as oxylipins, lipophilic vitamins, and plant-derived chemical analogs, are reported to be beneficial to human health and are thus widely accepted as therapeutic agents for the prevention and treatment of disease [8]. However, these observations are frequently empirical, and no rationale or mechanistic insight is available, highlighting the importance of filling the existing gap of knowledge in this field of study.

## 2. Bioactive Lipid Classes

### 2.1. Oxylipins

Oxylipins are oxidized bioactive lipids derived from polyunsaturated fatty acids (PUFAs), typically linoleic acid (for ω-6 fatty acids) and α-linolenic acid (for ω-3 fatty acids). These two PUFAs are known as the essential fatty acids (EFAs) because mammals cannot synthesize them, and they must be obtained from the diet. Starting with these two building blocks, it is possible for mammals to synthesize all the relevant downstream oxylipin PUFA precursors by elongation and desaturation reactions. Intermediates can also be acquired through the diet, which, in many cases, constitutes the main input source rather than endogenous synthesis (e.g., docosahexaenoic acid (DHA); [28]). The main three enzymatic oxidation pathways for oxylipin production involve cyclooxygenases (COXs), lipoxygenases (LOXs), and cytochrome P450 epoxygenase enzymes [29,30]. Non-enzymatic oxidation is also possible, for example, under conditions of high cellular oxidative stress, although its biological role is unclear in many cases. These reactions consequently lead to the formation of a host of bioactive lipids, such as prostaglandins, thromboxanes, mono-, di-, and tri-hydroxy fatty acids (FAs), epoxy FAs, lipoxins, eoxins, hepoxilins, resolvins, protectins, maresins, and other less characterized forms [31]. Oxylipins are important in autocrine and paracrine signaling and play crucial roles in the cardiovascular system in angiogenesis [32], blood vessel permeability [31], inflammation [33], and blood coagulation [34]. Despite this, many oxylipins are poorly understood and remain uncharacterized. Hundreds of new species have been discovered in the last two decades, and only a handful have been extensively studied [6].

**Figure 1:**
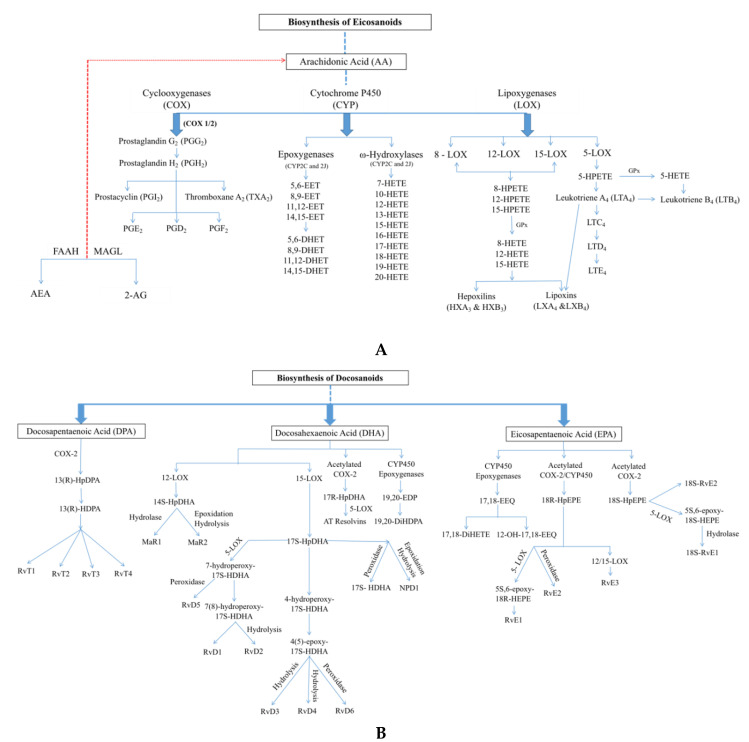
Biosynthesis of eicosanoids and docosanoids. (**A**) Eicosanoid biosynthesis starts with the release of arachidonic acid (AA) from membrane phospholipids and is catalyzed by three major enzyme families: cyclooxygenases (COXs), lipoxygenases (LOXs), and cytochrome P450 epoxygenases (CYPs). COXs produce prostaglandins (PGs) and thromboxanes (TXs) through the actions of specific synthases; LOXs produce biologically active metabolites, such as hydroperoxyeicosatetraenoic acids (HPETEs), hydroxyeicosatetraenoic acids (HETEs), and leukotrienes (LTs), while CYPs metabolize AA into epoxyeicosatrienoic acids (EETs), dihydroxyeicosatrienoic acids (DHETs), and HETEs. Arachidonic acid can be recycled from the breakdown of endocannabinoids through the actions of fatty acid amide hydrolase (FAAH) and monoacylglycerol lipase (MAGL). (**B**) Docosanoids originate from eicosapentaenoic acid (EPA), docosapentaenoic acid (DPA), and docosahexaenoic acid (DHA), which are then converted into a series of lipid mediators, including resolvins, protectins, and maresins. **Abbreviations:** 2-AG, 2-arachidonoylglycerol; AA, arachidonic acid; AEA, *N*-arachidonoylethanolamine/anandamide; AT, aspirin-triggered; COX, cyclooxygenase; CYP, cytochrome P450; DHA, docosahexaenoic acid; DHET, dihydroxyeicosatrienoic acid; DiHDPA, dihydroxydocosapentaenoic acid; DiHETE, dihydroxyeicosatetraenoic acid; DPA, docosapentaenoic acid; EDP, epoxydocosapentaenoic acid; EEQ, epoxyeicosatetraenoic acid; EET, epoxyeicosatrienoic acid; EPA, eicosapentaenoic acid; FAAH, fatty acid amide hydrolase; GPx, glutathione peroxidase; HDHA, hydroxydocosahexaenoic acid; HDPA, hydroxydocosapentaenoic acid; HEPE, hydroxyeicosapentaenoic acid; HETE, hydroxyeicosatetraenoic acid; HpDHA, hydroperoxydocosahexaenoic acid; HpDPA, hydroperoxydocosapentaenoic acid; HpEPE, hydroperoxyeicosapentaenoic acid; HPETE, hydroperoxyeicosatetraenoic acid; HX, hepoxilin; LOX, lipoxygenase; LT, leukotriene, LX, lipoxin; MAGL, monoacylglycerol lipase; MaR, maresin; NP, neuroprotectin; OH, hydroxyl group; PG, prostaglandin; RV, resolvin; TX, thromboxane.

*Eicosanoids*. The oldest known and best-characterized oxylipins are eicosanoids. The first prostaglandin was found in semen (and thought to be produced by the prostate gland, hence the name) [35] but was not identified until five years later [36]. Comprehensive identification and characterization of eicosanoids did not take place until the 1960s and 70s [37,38] when their anti-inflammatory properties and synthesis from arachidonic acid (AA) were demonstrated. AA can be obtained from the diet (animal tissues) but can also be synthesized from linoleic acid present in nuts and seeds [39]. Eicosanoids are then directly derived from their ω-6 PUFA precursor AA (and to a lesser extent dihomo-γ-linolenic acid) through the serial actions of cyclooxygenases (e.g., prostaglandins) and/or lipoxygenases (e.g., hydroxyeicosatetraenoic acids (HETEs))—two specialized families of oxygenation enzymes. Epoxyeicosatrienoic acids (EETs), a subfamily of eicosanoid-like molecules, can also be produced by the action of cytochrome P450 epoxygenase enzymes (see Figure 1A). Eicosanoid-derived oxylipins are involved in cardiac injury and dysfunction [40], atherosclerosis [41], blood clotting and platelet aggregation [42,43], and blood pressure regulation [44].

Eicosanoids are produced close to their site of action and are then quickly degraded, limiting their actions to paracrine/autocrine functions [42]. There are three classical types of eicosanoids: prostaglandins, thromboxanes, and leukotrienes [45]. Prostaglandins (PGs) play crucial roles in the early stages of inflammation (e.g., PGE_2_, PGI_2_) but also in tissue homeostasis, cancer, and stem cell biology [46,47]. Thromboxanes (TXs) generally serve as platelet agonists during blood clot formation [48]. Leukotrienes (LTs) are involved in the inflammatory response and the progression of atherosclerosis due to their roles in endothelial dysfunction and cytokine release [49]. Eicosanoids have the ability to regulate vascular function through GPCRs in endothelial cells (ECs) and vascular smooth muscle cells (VSMCs) and also control barrier formation, platelet aggregation, and mucosal integrity in the stomach [42,50,51]. AA itself has been involved in cellular signaling as a lipid second messenger, helping to regulate the activity of many signaling enzymes, including isoforms of the protein kinase C (PKC) and phospholipase C (PLC) families [52]. AA is frequently connected with protection from heart disease [51], control of vascular tone [53], and platelet aggregation [54].

*Docosanoids*. Docosanoids or specialized pro-resolving mediators (SPMs) are endogenous bioactive lipid mediator molecules derived from α-linolenic acid or its omega-3 PUFA derivatives—eicosapentaenoic acid (EPA), docosapentaenoic acid (DPA), and docosahexaenoic acid (DHA)—by the action of cyclooxygenases, lipoxygenases, and epoxygenases [55,56,57] (see Figure 1B). Docosanoid precursor molecules are mostly obtained from the diet and are present in significant amounts in various plants and nuts (e.g., camelina, flaxseed), fish, seafood, phytoplankton, and marine seaweeds [58]. Docosanoids were discovered in the early 2000s by Serhan et al. in inflammatory exudates and possess potent activity in the resolution of inflammation and restoration of tissue homeostasis [59,60,61]. They can also influence a wide range of other processes, including cardiac rhythm [62], smooth muscle contraction [63], platelet reactivity [64], and blood clotting [65]. Docosanoids modulate several biological activities related to the heart, including lowering of blood triglyceride levels, antiarrhythmic effects, modulation of the eicosanoid system towards vasodilatation, and lowering of pro-atherogenic cytokine levels [66,67]. The docosanoid family includes several structurally distinct classes, including resolvins, protectins, and maresins (some of which are still expanding due to the quick and ongoing discovery of new members). The best-studied docosanoid class is the resolvin E family. Series-E resolvins (RvEs) are EPA-derived metabolites synthesized by acetylated COX-2 or a cytochrome P450 enzyme. This process occurs mainly when endothelial cells interact with leukocytes during inflammation processes. There are three sub-types of RvEs that have been described in the literature: RvE1 (5S,12R,18R-trihydroxy-6Z,8E,10E,14Z,16E-EPE), RvE2 (5S,18R-dihydroxy-6E,8Z,11Z,14Z,16E-EPE), and RvE3 (17R,18R/S-dihydroxy-5Z,8Z,11Z,13E,15E-EPE). The mechanism of action of these compounds appears to be mediated by activation of extracellular signal-regulated kinase 1/2 (ERK1/2) and phosphoinositide 3-kinase (PI3K)/Akt signaling pathways, activation of endothelial nitric oxide synthase (eNOS) by phosphorylation, and reduction of caspase-3 activity [68,69,70]. In addition, members of the RvD family (RvD1 and RvD2) are reported to have positive effects in many processes and organ pathologies, including wound healing [71], fibrosis [72,73], and vascular disease [74]. The bioactivity of resolvins and protectins has been verified by the chemical synthesis of pure compounds. CVD often presents with inflammation, and as such, it is highly likely that resolvins and protectins might play important roles in this setting.

Besides serving as substrates for the synthesis of all docosanoids, EPA, DPA, and DHA constitute essential structural molecules that contribute to cell membrane fluidity (similar to the role played by AA in membrane phospholipids). EPA and DHA get incorporated into the cell membrane phospholipids of diverse cell types, including cardiac cells [75], endothelial cells [76], platelets, and immune cells. DPA is implicated in platelet aggregation, plasma lipid parameters, cellular plasticity, and amelioration of cardiovascular and metabolic disease risk. DPA is the intermediate fatty acid species between EPA and DHA and may also play a role in impacting the health benefits previously attributed solely to EPA and DHA [77]. EPA, DPA, and DHA are important dietary supplements for pregnant mothers as they help to control infant birth weight and fetal and neonatal neurodevelopment [78]. Mixtures of EPA, DPA, and DHA are also strongly and significantly associated with a lower risk of CHD [79]. In the early 2000s, the American Heart Association, the European Society for Cardiology, and other international cardiac societies recommended the intake of one gram per day of the two marine omega-3 fatty acids (EPA and DHA) for secondary prevention of cardiovascular disease, treatment post-myocardial infarction, and prevention of sudden cardiac death [80,81]. The biochemical and physiological mechanisms leading to these benefits remain incompletely understood but seem to center around positive effects on the vasculature that counteract those of omega-6 fatty acids [82,83,84].

### 2.2. Lysophospholipids and Sphingolipids

*Lysophospholipids*. Lysophospholipids constitute a class of bioactive lipids with numerous biological functions during cellular differentiation and development [85]. They are normally obtained from natural sources, such as egg yolk or soybean, and enzyme-catalyzed synthesis starts from these phospholipids [86]. Lysophospholipids are divided into two general categories: sphingosyl-based lysophospholipids (lysosphingolipids) and glyceryl-based lysophospholipids (lysoglycerophospholipids), which have either sphingosine or a glycerol backbone, respectively [87]. Some key members of the lysophospholipid group are lysophosphatidic acid (LPA), sphingosylphosphorylcholine (SPC), lysophosphatidylcholine (LPC), lysophosphatidylinositol (LPI), and sphingosine-1-phosphate (S1P), which can also be considered a sphingolipid. They are all highly conserved among vertebrates. The biological activity of lysophospholipids is accomplished fundamentally through the activation of G protein-coupled receptors, although other mechanisms have been described (e.g., activation of transient receptor potential (TRP) channels). The endothelial differentiation gene (EDG) family (LPA receptors 1-3) and non-EDG family (LPA receptors 4-6) constitute a specialized family of GPCRs dedicated to lysophospholipid signaling. LPARs carry downstream signals through at least four heterotrimeric Gα proteins (Gαq/11, Gα12/13, Gαi/o, and Gαs) in different physiological and pathological conditions [88,89]. LPA (a major lysoglycerophospholipid) is mobilized through multiple enzymes, including extracellular phospholipase A2 (PLA2), lysophospholipase D (lysoPLD), and monoacylglycerol kinase (MAG kinase). Likewise, the levels of extracellular LPA are regulated by the actions of many enzymes, including three lipid phosphate phosphatases (LPPs), LPA acyltransferase, and lysophospholipases [90]. Peroxisome proliferator-activated receptor γ (PPARγ) and the P2Y purinoceptor 10 (P2Y10) have also been reported to mediate LPA signaling [91,92]. Lysophosphatidic acid has physiological and pathological actions in cell-to-cell interactions, cell proliferation and differentiation, cytoskeletal rearrangement, and tumorigenesis in various systems [93,94,95]. Dysregulation in LPA signaling has been discussed as the cause of disorders, such as atherosclerosis [96], inflammation [97], obesity [98], autoimmune diseases [99], cancer [100], and neuropathic pain [101]. LPA also has protective effects on the cardiovascular system through activation of downstream pathways involved in Gi-coupled signaling and PI3K/AKT and ERK activity. Targeted deletion of LPA receptors has uncovered essential roles for LPA signaling in the development and maintenance of heart rate [102], the formation of atherosclerotic plaques [103], and maturation of blood vessels [104,105,106]. Much of the recent research regarding LPA effects in the cardiovascular system has focused on the vasculature. LPA influences nearly all types of vasculature cells, including platelets, monocytes, macrophages, dendritic cells, vascular endothelial cells, T-lymphocytes, vascular smooth muscle cells, and fibroblasts [107]. However, the exact receptor that initiates these effects in different vasculature cells is still unknown. LPA activation of Edg and P2Y results in embryonic blood vessel formation [89,108]. In addition, several in vitro reports have demonstrated that the impact of LPA and its receptors in the vasculature includes elevated adhesion molecule expression, enhanced cell proliferation and migration, cytokine secretion, and increased chemokine levels [96,107,109]. Furthermore, in vivo studies of exogenous administration of LPA in rodents and different mammalian species show an increase in blood pressure and acute vasoconstriction, and local infusion of LPA in carotid arteries induces progressive vascular neointima formation [107,110,111].

*Sphingolipids*. Sphingolipids are derived from L-serine and fatty acid, which are collectively defined as a “sphingoid” base, also called a long-chain base (LCB). The initiation of de novo biosynthesis occurs via the condensation of palmitoyl-CoA and serine, which is catalyzed by the rate-limiting enzyme serine palmitoyltransferase (SPT), resulting in 3-ketosphinganine (3-ketodihydrosphingosine). In the presence of reduced nicotinamide adenine dinucleotide phosphate (NADPH), this ketone intermediate can then be reduced to the lysosphingolipid sphinganine, which serves as a precursor for many bioactive sphingolipids. Lysosphingolipids are sphingolipids with a sphingoid base that lacks an N-acyl fatty acid [112]. In general, several sphingolipids act as cell adhesion molecules and regulate cell proliferation via signal transduction pathways involving growth factor receptor phosphorylation/dephosphorylation and NADPH oxidase–mediated superoxide generation. These signaling pathways are initiated by tyrosine kinase receptors and activate PKC, mitogen-activated protein kinase (MAPK), MEK, Raf-1, and other kinases and/or alter cytosolic Ca+ levels, leading to changes in cell proliferation, differentiation, and apoptosis. In the cardiovascular system, sphingolipids are mostly involved in cardiogenesis [113], heart maturation [114], angiogenesis [115], inflammation [116], blood vessel permeability [117], and regulation of vascular tone [118]. It has been observed that sphingolipids exert a role in cardiovascular development by inducing the maturation of the mouse embryonic vascular system and altering cardiac morphology in zebrafish and mouse embryos [119]. Sphingolipids and their derivatives are linked to many embryonic stem (ES) cell biological processes, including the promotion of cell survival through proliferation, the regulation of gene expression related to pluripotency, differentiation, inflammation, apoptosis, cell cycle regulation, cell polarity, and migration [120,121]. Specific members of the sphingolipid family include sphingosine (Sph), SPC, and S1P (also considered lysophospholipids), ceramide (Cer), and ceramide-1-phosphate (C1P) [122]. Five G protein-coupled receptors are known for S1P (a major lysosphingolipid), named S1P_1-5_, and S1P can act both extracellularly through these receptors and intracellularly through direct interactions with target proteins [123]. These receptors function in the cardiovascular, nervous, immune, and reproductive systems [124]. S1P and its GPCRs may play critical roles in cardiovascular and immune development and function [125]. S1P acts on hematopoietic progenitor cells as a chemotactic factor and has also been shown to be critical for cardiac and skeletal muscle regeneration [9]. It may also contribute to the protective effects seen in the heart during ischemic challenge induced by myocardial infarction [126]. Interestingly, S1P may act to maintain human stem cell pluripotency, as both LPA and S1P positively regulate the proliferative capacity of murine ES cells [9]. SPC induces mesodermal differentiation of mouse ES cells and differentiation of promyelocytic leukemia cells by a mechanism critically dependent on MEK-ERK signaling [127]. SPC and S1P are both able to promote the differentiation of umbilical cord mesenchymal and Sca1+ cardiac stem cells to cardiomyocytes [128]. They can also enhance induced pluripotent stem cell differentiation to cardiomyocytes at an early stage of differentiation [17]. Additionally, S1P can support the differentiation of human adipose tissue mesenchymal stem cells (hATMSCs), human umbilical cord mesenchymal stem cells (hUCMSCs), and mesenchymal stem cells (MSCs), as it stimulates hATMSCs dose-dependently, leading to differentiation of smooth muscle cells.

### 2.3. Endocannabinoids

In 1964, Israeli scientists Mechoulam and Ganoi first extracted the cannabinoid (CB) molecule tetrahydrocannabinol (THC) from *Cannabis sativa*, an herbaceous flowering plant. According to Mechoulam, endocannabinoids (ECs) can be regarded as an “overall protective network, several physiological systems, and working in concurrence with the immune system” [129,130]. ECs are highly conserved endogenous bioactive lipid mediator molecules associated with a range of physiological and pathological conditions in many organs. The two most well-characterized ECs are anandamide (AEA) and 2-arachidonoylglycerol (2-AG). AEA is derived from the N-arachidonoylphosphatidyl-ethanolamine (NArPE) family of glycerophospholipids through phospholipase-mediated reactions, and 2-AG is derived from membrane phospholipids through the activity of phospholipase C, with diacylglycerol (DAG) as an intermediate [131]. Fatty acid amide hydrolase (FAAH) and monoacylglycerol lipase (MAGL) are the major enzymes responsible for the fast turnover of these ECs in cells. FAAH metabolizes AEA to free AA and ethanolamine, and MAGL metabolizes 2-AG to free AA and glycerol. ECs and their receptors are present in humans and animals, such as mammals, fish, earthworms, amphibians, reptiles, leeches, and birds (all except insects). They are widely dispersed in the central nervous system (CNS), peripheral tissues, and immune systems of mammals, where they have several roles, including the regulation of cognitive functions [132] and neuroprotection [133]. ECs act through different receptors but prominently interact with G-protein coupled receptor (GPCR) family cannabinoid receptors 1 (CB1R) and 2 (CB2R). CB1 receptors are abundant in different regions of brain presynaptic terminals, such as the hippocampus, substantia nigra, cerebral cortex, and striatum [134]. These receptors are also present in peripheral regions, such as the adrenal gland, reproductive tissues, and immune cells, at lower levels [135]. CB2 receptors are mainly expressed in the immune organs, including the spleen, thymus, and lymph nodes, and are involved in the immunomodulatory effects of cannabinoids [136]. Activation of the CB receptors triggers intricate cascades of intracellular signaling molecules, cellular functions, electrical excitability of cell membranes, and gene expression. Other major receptors that mediate the biological effects of ECs include G-protein coupled receptor 18 (GPR18), GPR55, GPR92, GPR119, and PPARα. AEA and 2-AG are found in diverse parts of the cardiovascular system, including the myocardium, vascular cells, platelets, red blood cells, and serum. Endocannabinoids have several protective roles in various pathophysiological conditions, especially in CVD [137]. In fact, the endocannabinoid system (ECS) may play a pivotal part in therapeutic potential in several cardiovascular diseases, including myocardial infarction, circulatory shock, cerebral ischemia, arrhythmias, hypertension, atherosclerosis, and metabolic disorders. The expression of CB1R has seemingly little-to-no effect on cardiac disease. Although CB2R expression has been less well-characterized, it has been confirmed in the cardiovascular system and has shown cardioprotective and anti-inflammatory activity and necessitates further research.

### 2.4. Steroids and Lipid-Soluble Vitamins

*Steroids*. Steroids are a heterogeneous group of organic molecules that act in the body as hormones [138]. They are generally grouped into two categories—corticosteroids (glucocorticoids and mineralocorticoids) and sex steroids (androgens, estrogens, and progestogens)—with vitamin D and its derivatives often included as a third category [139,140]. Vitamin D comes in two major forms—vitamin D_2_ (ergocalciferol) and vitamin D_3_ (cholecalciferol). The skin synthesizes vitamin D_3_ after sun exposure (specifically UVB radiation), and it may also be obtained from animal sources, such as fish. Vitamin D_2_ is the synthetic form that is often found in food supplements and can be obtained from plants [140]. The vitamin D synthesized by the skin or obtained through the diet is an inactive form, as it must first be transported to the liver to form calcifediol and then circulates to the kidney tubules to form calcitriol, the most active hormonal form of vitamin D [140,141,142,143]. Active calcitriol travels to the nuclei of target tissues and binds to the vitamin D receptor (VDR), which can then act as a transcription factor to increase the expression of genes involved in ion binding, transport, and absorption [140,144]. When no longer needed, the metabolites of vitamin D are hydroxylated by the CYP24A1 enzyme in order to initiate their degradation [145,146]. A lack of exposure to sunlight, as well as a diet insufficient in vitamin D intake, can lead to deficiency, and it is estimated that one billion people worldwide fall under this classification [147,148]. While the primary conditions resulting from vitamin D deficiency are related to bone health (osteomalacia and rickets), there is also evidence that an insufficiency can increase the incidence of cardiovascular disease [147]. Studies suggest that low serum levels of 25-hydroxyvitamin D lead to an increased risk for MI in healthy individuals [149] and an increased risk for cardiovascular mortality in individuals with coronary heart disease [150]. However, this evidence is inconclusive at best, and there is still a question as to whether vitamin D intake can actually prevent CVD [151,152]. Exposure to high doses of steroids other than vitamin D is generally considered detrimental to the cardiovascular system. Glucocorticoids are potent regulators of stress responses and, as such, are implicated in causing hyperglycemia [153], hypertension [154], dyslipidemia [155], and central obesity [156,157]. There is also evidence that oral glucocorticoid administration increases the risk of heart failure almost three-fold compared to non-users [158], potentially through effects on the mineralocorticoid receptor [159,160]. However, corticosteroids also have potent anti-inflammatory effects and, as such, may be beneficial in atherosclerosis patients [161,162]. In addition, glucocorticoids are essential for maturing fetal heart structure and function [163,164].

**Figure 2:**
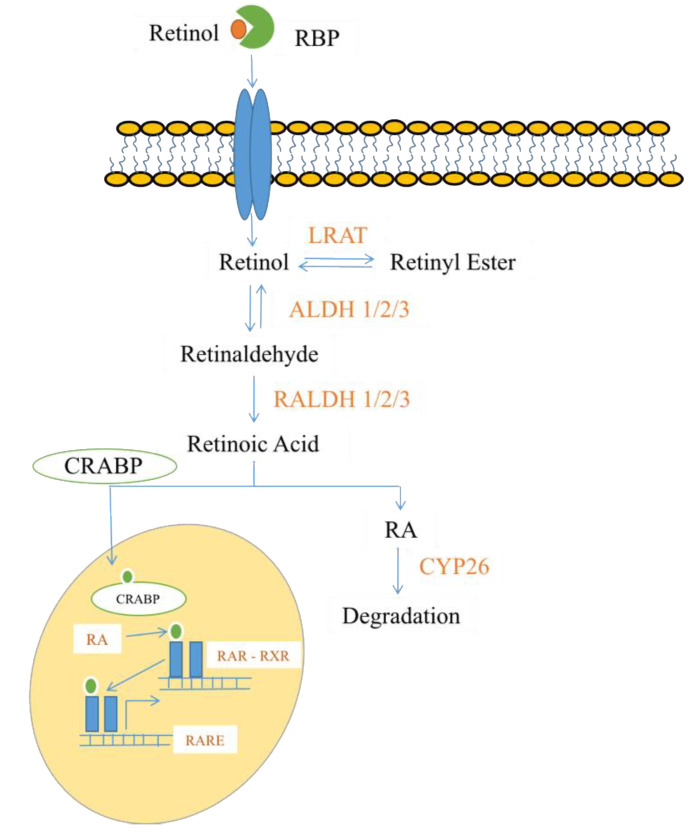
Mechanism of retinoic acid signaling. Upon reaching its target tissue, retinol is released from RBP and enters cells through specialized receptors. Once inside the cell, retinol can be stored as retinyl esters or irreversibly metabolized to retinoic acid by retinaldehyde dehydrogenase enzymes through a retinaldehyde intermediate. Bioactive RA enters the nucleus bound to CRABP and activates a RAR-RXR heterodimer, leading to transcription of RAREs. When no longer needed, RA is degraded by CYP26 enzymes and cleared from the body. **Abbreviations:** ALDH, aldehyde dehydrogenase; CRABP, cellular retinoic acid-binding protein; CYP26, family 26 of cytochrome P450 enzymes; LRAT, lecithin retinol acyltransferase; RA, retinoic acid; RALDH, retinaldehyde dehydrogenase; RAR, retinoic acid receptor; RARE, retinoic acid response element; RBP, retinol-binding protein; RXR, retinoid X receptor.

*Retinoids*. Retinoids are a family of nutritional bioactive lipids, commonly known as vitamin A. Three of the most common retinoids are retinol (the predominant form obtained through food), retinal, and retinoic acid (RA) [165,166]. They are essential for normal growth, reproduction, immunity, and vision [167,168]. Retinoids were first observed in 1816 by Magendie, who noted that nutritionally-deprived dogs displayed high levels of corneal ulcers and mortality, but they were not structurally characterized until 1931 [169,170]. The breakdown of beta-carotene, a terpenoid compound found in plants and fruits, yields two molecules of vitamin A. This process occurs in the small intestine, and the resulting retinol molecules can be stored as retinyl esters in the liver. Alternatively, these compounds can be converted to retinal, an active form of vitamin A [171]. Retinal is an important pigment molecule involved in phototransduction, as it undergoes an isomerization reaction (conversion of the *cis* form to the *trans* form) upon exposure to photons of light [172]. If necessary, retinal can be further metabolized to retinoic acid in an irreversible reaction catalyzed by retinaldehyde dehydrogenase (RALDH) enzymes. When in the cell cytoplasm, RA is bound to the cellular retinoic acid-binding protein (CRABP). When needed, RA enters the nucleus and binds to a heterodimer consisting of a retinoic acid receptor (RAR) and a retinoid X receptor (RXR). This heterodimer then binds specifically to a DNA sequence, known as a retinoic acid response element (RARE), in order to regulate gene transcription [173]. If cells no longer need RA signaling, members of the cytochrome P450 family of enzymes (particularly CYP26) can metabolize and clear it from the body [174,175] (see Figure 2 for an overview of RA signaling mechanisms). Retinoic acid is a fundamental morphogen in embryonic tissues and organs during early development, with patterning functions affecting organs, such as the brain, heart, eyes, gonads, and lungs [165,176]. Excessive or inappropriate signaling of RA in embryonic development reveals complex structural abnormalities and malformations of the fetus. RA has important functions in stem cell proliferation and cell differentiation [177] and is required for the formation of cardiac progenitor cells and the correct establishment of the first and second heart fields [178,179,180]. Indeed, a lack of proper retinoic acid signaling in embryonic development has been shown to cause perinatal lethality in the majority of mice [181]. Those that survive display a phenotype similar to that seen in human DiGeorge syndrome (DGS)—a genetic condition characterized by cardiac conotruncal malformations, aortic arch abnormalities, and facial and thyroid developmental defects [182]. While the effects of retinoids on the cardiovascular system are most well-characterized in development, there is also evidence that RA may be beneficial in preventing coronary artery disease in mice [183] and reducing its mortality rates in humans [184].

## 3. Recent Advances in Bioactive Lipids in Cardiac Disease

### 3.1. Cardioprotective Oxylipins

The potential benefits of oxylipins in the eventual treatment of cardiovascular disease are readily apparent in clinical trials conducted using human volunteers. In an early study, Grimsgaard et al. [185] showed that EPA and DHA administration lowered serum triacylglycerol levels, with differential effects on cholesterol levels. In a more recent trial, individuals presenting with symptoms of acute myocardial infarction (MI) were randomly assigned to receive high-dose omega-3 fatty acids (EPA and DHA) or a placebo capsule (corn oil) four times daily for six months. After completion of the trial, treated patients showed higher omega-3 fatty acid levels in red blood cells, a reduction in left ventricular end-systolic volume indexed to body surface area (LVESVI, indicative of adverse ventricular remodeling), and a reduction in myocardial fibrosis compared to the placebo group. In addition, there was a slight increase in left ventricular ejection fraction (LVEF) and a significant decrease in the levels of serum biomarkers associated with myocardial dysfunction (ST2, Lp-PLA2, triglycerides) [186]. Another recent clinical trial further enforced these findings, this time looking at the incidence of ischemic events in CVD patients with elevated triglyceride and cholesterol levels. Enrolled patients were randomly assigned to receive 2 g of icosapent ethyl (a purified form of EPA) or placebo twice daily for approximately five years. Upon follow up, the treated patients displayed a reduction in serum triglyceride levels and a lower increase in low-density lipoprotein (LDL) levels compared to the placebo group. An important endpoint for an individual enrolled in this study was cardiovascular death, MI, or stroke, and only 11.2% of treated patients experienced one of these events compared to 14.8% in the placebo group. These differences remained in place when also considering coronary revascularization and angina occurrence, thus demonstrating the importance of oxylipins in preventing ischemic events in individuals with CVD [187]. Still, another recent human study quantified the effects of specialized pro-resolving mediators, specifically DPA-derived resolvins, in CVD. The authors measured plasma levels of D-series resolvins (RvD_n-3 DPA_) in healthy volunteers and discovered that they increased throughout the night and decreased throughout the day. Individuals with CVD displayed an alteration in these fluctuations and lower overall DPA-derived resolvin levels, which were associated with higher levels of leukocyte activation and inflammation. Notably, the incubation of patient peripheral blood samples with RvD_n-3 DPA_ reduced leukocyte and platelet activation, suggesting that dysregulation of these resolvins might lead to CVD [188].

In addition to the above clinical trial data, there is also an abundance of evidence supporting a role for oxylipins in cardiovascular disease. A recent in vitro study has shown that a combination of EPA and DHA along with ascorbic acid (AA) has a protective effect on ES cell-derived cardiac progenitor cells. Incubation with this bioactive lipid cocktail before hydrogen peroxide treatment increases the expression of cardiomyocyte markers, decreases the expression of fibroblast markers, and reduces the production of harmful reactive oxygen species. It is also notable that these oxylipins reduce the accumulation of fibrosis after myocardial infarction in ischemic rat hearts [189]. One particular area of study that is generating much interest is the beneficial pro-resolution of inflammation mediated by docosanoids. A recent report by Kain et al. [190] utilized two forms of resolvin D1 (RvD1)—one incorporated into liposomes and one in a free acid form–to study its effects post-MI. Adult mice were subjected to coronary artery ligation, and 3 µg/kg resolvin was injected subcutaneously three hours post-injury. Five days post-MI, the RvD1-treated groups showed a decreased left ventricular mass to body weight ratio and increased fractional shortening ratio compared to controls, indicative of an improvement in left ventricular function. Interestingly, neutrophil levels in the left ventricle peaked at day 1, and by day 5, the treated animals already displayed reduced neutrophil recruitment to the injury site and lower macrophage density in the left ventricle. In addition, the levels of SPMs (resolvins, maresins, and lipoxins) were increased in the mouse spleens, and the expression of the lipoxin A_4_ receptor and 5-lipoxygenase enzyme was higher compared to saline-treated mice. These findings were all consistent with resolvin administration promoting the resolution of the acute inflammatory phase post-MI [191]. It is also notable that RvD1 treatment reduced collagen deposition and the expression of extracellular matrix genes in the infarcted area, which further supports it having a cardioprotective role post-MI, possibly by delaying the onset of heart failure [190]. This same group later conducted two similar studies with similar results, but this time through the administration of 15-epi-lipoxin A_4_ in liposomes [192] and by genetic deletion of 12/15 lipoxygenase enzymes and subsequent MI induction [193]. Recent findings of the benefits of bioactive lipid-mediated inflammatory resolution in cardiovascular disease do not just apply to MI but also to vascular disorders. Fredman et al. [194] utilized targeted mass spectrometry to identify SPMs in human carotid atherosclerotic plaques and found that the ratios of pro-resolving RvD1 to pro-inflammatory leukotriene B_4_ were significantly decreased in vulnerable plaque regions compared to stable regions. Plaques isolated from low-density lipoprotein receptor knockout (*Ldlr^-/-^*) mice also showed similar lipid ratios, and intraperitoneal administration of 100 ng RvD1 three times per week for five weeks normalized this ratio. In addition, resolvin treatment decreased oxidative stress, fibrosis, and necrosis levels in the plaques and led to the production of thicker fibrous caps compared to vehicle-treated animals, thus demonstrating that the resolution of inflammation could have a beneficial effect in promoting plaque stability in individuals with atherosclerosis [194]. There is also recent evidence that the administration of resolvin D4 improves thrombus resolution and, therefore, reduces the severity of deep vein thrombosis (DVT) in mice [195]. All of the above reports demonstrate how critical the resolution of inflammation is in mitigating the severity of CVD and implicate bioactive lipids as potential therapeutic options for activating this resolution process.

The beneficial effects of bioactive lipids on CVD are not just attributable to resolvins as several different classes of eicosanoids and docosanoids have been linked to cardioprotection in the last 10–20 years. Administration of 19-hydroxyeicosatetraenoic acid (19-HETE) to adult rats ameliorates the hypertrophic phenotype of the heart induced by angiotensin II (lower left ventricular wall thickness and heart weight to tibia length ratio). In addition, 19-HETE decreases the levels of cardiac hypertrophy genes in human cardiomyocytes [196]—findings that are mostly attributable to the S-enantiomer [197]. 19-HETE also plays a role in the vasculature as treatment reduces platelet aggregation and facilitates mouse vessel relaxation—effects that are dependent on its acting through the prostaglandin I_2_ receptor [198]. HETEs are not the only class of eicosanoids with cardioprotective activity. Cytochrome P450 2J2 (CYP2J2) is an epoxygenase enzyme that mediates the formation of epoxyeicosatrienoic acids (EETs) [199], and overexpression of this enzyme in mouse hearts suppresses cardiac hypertrophy and increases levels of atrial natriuretic peptide (ANP) [200]. CYP2J2 overexpression in *Apoe^-/-^* mice also prevents atherosclerosis induced by a high-fat diet [201]. Interestingly, both of these studies have similar mechanistic findings and demonstrate that Akt (PKB) is activated downstream of CYP2J2 and EETs. Soluble epoxide hydrolases (sEHs) are enzymes that break down EETs [202], and inhibitors of these enzymes have been shown to prevent experimental ischemia-reperfusion injury in normal and hypertensive rat heart isolates [203]. Inhibition of sEH in rats with pulmonary hypertension also decreases pressure in the right ventricle and reduces pulmonary artery wall thickness, thereby demonstrating that maintenance of EET levels may be beneficial for the prevention of multiple different types of CVD [204]. One class of SPMs that has garnered interest in recent years is the lipoxins. Aspirin-triggered lipoxins (ATLs) are anti-inflammatory molecules produced by acetylated cyclooxygenase-2 enzymes, a process that is mediated by aspirin [205]. A recent study indicates that after four weeks of continuous ATL A4 infusion, *Apoe^-/-^* mice on a high-fat diet display reduced numbers of atherosclerotic lesions in the aorta. Treated animals also have lower levels of inflammatory molecules in the aorta, implying a resolution of inflammation [206]. Evidence suggests that ATLs act through the formyl peptide receptor 2 (FPR2)/lipoxin A4 receptor (ALX) to exert their effects, as intimal hyperplasia after carotid artery ligation is reduced by ATL infusion but only in mice that express this receptor [207]. ATL signaling through FPR2/ALX may also protect against abdominal aortic aneurysms [208]. In addition to lipoxins, protectins [209] and maresins [210] are also beneficial in the vasculature, although further research is required to establish mechanistic links. Clearly, the potential cardioprotective effects of oxylipins are numerous, and many different classes play a role in mediating these benefits.

### 3.2. Deleterious Effects of Oxylipins

It is important to note that not all clinical trials unanimously suggest that oxylipins are beneficial in preventing cardiovascular disease. Caligiuri et al. [211] recently discovered that consumption of flaxseeds by patients with peripheral artery disease significantly reduced central blood pressure. These effects might be mediated through oxylipins, as a 1 nM increase in 16-HETrE, 5,6-diHETrE, and TXB_2_ led to at least a 10-fold increase in blood pressure. Indeed, flaxseed consumption lowered the levels of these lipids, thereby demonstrating that an effective hypertension treatment might involve reducing oxylipin levels in the body [211]. In fact, some oxylipins have a deleterious effect on cardiovascular disease, as several reports show a correlation between linoleic acid (LA) intake and coronary disorders. The consumption of seed oils high in LA contributes to inflammation, oxidative stress, endothelial dysfunction, and atherosclerosis [212,213]. One pro-inflammatory class of eicosanoid that has been implicated in CVD in preclinical studies is the leukotrienes. In a recent report, de Hoog et al. [214] subjected mice to myocardial ischemia for 30 min by coronary artery ligation and followed by allowing the injured area to reperfuse. Half of the mice were then treated with 50 mg/kg LSN2792613, a selective BLT1 (leukotriene B_4_ receptor) antagonist, three times daily by oral gavage. Twenty-four hours after ischemia-reperfusion injury, treated mice displayed a significant reduction in infarct size, inflammatory cell (neutrophils, macrophages, T cells) and inflammatory cytokine levels in the infarct region, and percentage of cells undergoing apoptosis, relative to controls. While the effects on cardiac function were minimal, these results suggested that inhibition of leukotriene signaling reduced inflammation following ischemia-reperfusion injury, which might be a promising way to treat individuals who have experienced MI [214]. Similar findings have also been obtained for leukotriene C_4_, as pharmacological inhibition of its receptor (Cys-LT1) following cardiac cryoinjury leads to increased ejection fraction and reduced akinetic myocardial mass, thereby demonstrating a reduction of adverse myocardial remodeling [215]. Other recent studies suggest the detrimental effects of leukotrienes in vascular diseases. Endotoxemia is a condition where the increased release of lipopolysaccharide (LPS) into the bloodstream can lead to harmful plaque formation and increased risk of atherosclerosis [216]. Endotoxemia was induced in *Apoe^-/-^* mice by intraperitoneal injection of LPS for five consecutive days, and atherosclerotic plaques isolated from these mice produced 2.5-fold more leukotriene B_4_ than non-treated plaques. Treated plaques also showed higher levels of neutrophil infiltration and decreased stability—effects that were reversed by genetic and pharmacological inhibition of LTB_4_. Taken together, these data demonstrate that pro-inflammatory leukotrienes can induce plaque destabilization in conditions of endotoxemia, potentially exacerbating atherosclerosis and increasing the severity of cardiovascular disease [217]. Refer to Table 1 for a list of specific oxylipins discussed in this review and their functions in CVD.

### 3.3. Other Bioactive Lipids Involved in Cardiac Function

Over the last few years, endocannabinoids (ECs) have also been raised as a therapeutic target for a variety of disorders associated with tissue injury and inflammation, including those in the cardiovascular system. EC expression is tightly regulated in the circulation and in various tissues by connected synthases, transporters, and degrading enzymes [218]. ECs play a vital protective role in ischemia grafting and cardiovascular regulation associated with hypertension [219,220,221]. Steffens et al. experimentally showed that atherosclerosis development could be attenuated using 9-Δ-tetrahydrocannabinol, which interacted with CB2 receptors, causing inhibition of the inflammatory response [222,223]. In addition, anandamides dose-dependently decrease the expression of tumor necrosis factor- α (TNF-α)-induced intercellular adhesion molecule 1 (ICAM-1) and vascular cell adhesion molecule 1 (VCAM-1) and also reduce NF-κB activation in human coronary artery endothelial cells in a manner dependent on the CB1 and CB2 receptors [224]. These data suggest that inhibiting the fatty acid amide hydrolase (FAAH) enzyme, which metabolizes anandamide, may reduce chronic inflammation and oxidative stress in cardiovascular disease. Cannabidiol (CBD) is a plant-derived cannabis compound reported to have several beneficial effects in preclinical disease models of CVD (cardiomyopathies, myocardial infarction, arthritis), epilepsy, stroke, diabetes, inflammation and autoimmunity, kidney disease, neurodegenerative disorders, and cancer [225,226,227]. It is notable that CBD ameliorates cardiac function and decreases apoptosis of cardiomyocytes and endothelial cells by reducing nitrative and oxidative stress [228]. Several recent reports suggest that some steroids also have beneficial effects on CVD phenotypes. In humans, prenatal exposure to glucocorticoids improves cardiovascular function in the newborn immediately after birth [163,229,230,231,232]. Antenatal corticosteroid therapy (ACT) of a potent synthetic glucocorticoid mimics the activity of endogenous glucocorticoids and is prescribed to women to decrease the risk of preterm delivery and to mature fetal organs, including the heart [233]. The goal of ACT is to improve the survival of preterm infants and decrease neonatal morbidity. One recent in vitro study demonstrates that cardiomyocyte-specific deletion of the glucocorticoid receptor in mouse hearts causes them to develop cardiac hypertrophy and left ventricular systolic dysfunction, eventually progressing to heart failure. These pathologies are attributed to impaired calcium dynamics, oxidative stress, and higher levels of apoptosis when glucocorticoid signaling is not present [234]. Another report shows that glucocorticoids induce a dramatic maturation of cardiomyocyte (CM) myofibrillar content and organization in vitro. When applied to primary fetal mouse cardiomyocytes, corticosterone (a glucocorticoid) promotes myofibril growth and Z-disc assembly, improves CM contractility, and increases mitochondrial activity, all of which are indicative of enhanced CM maturity. The authors conclude that glucocorticoids induce expression of PGC-1α (a PPAR-gamma receptor coactivator) in order to mediate the maturation of fetal cardiomyocytes [164]. Although the effects of glucocorticoids in CVD still necessitate further research, early signs point to them playing a pivotal role in cardiomyocyte development, and, as such, they may be promising targets in the eventual treatment of congenital heart diseases.

There is also increasing evidence from the last several years that sphingolipids have a detrimental effect on cardiovascular function. Glycosphingolipids, which are merely sphingolipids with attached carbohydrate groups [112], have received a particular amount of attention for their involvement in the pathogenesis of atherosclerosis. A recent report by Chatterjee et al. [235] utilized *Apoe^-/-^* mice fed with a high fat/high cholesterol diet for several months to induce atherosclerosis. Half of the mice were given daily treatments of D-threo-1-phenyl-2-decanoylamino-3-morpholino-1-propanol (D-PDMP), a compound that inhibits glycosphingolipid synthesis, by oral gavage. Echocardiography measurements in the aorta after 20 and 36 weeks of treatment showed a dose-dependent decrease in pulse wave velocity (PWV, a measure of vascular stiffness), intima-media thickness (IMT, a measure of arterial wall thickness), atherosclerotic plaque buildup, and calcium deposits compared to placebo-treated animals. Serum cholesterol profile was also improved by D-PDMP treatment through increases in *Srebf2* and *Ldlr* expression (to increase LDL metabolism) and ATP-binding cassette (ABC) transporter expression (to increase cholesterol efflux from peripheral tissues for excretion). Interestingly, similar results were obtained when atherosclerotic rabbits were fed D-PDMP [235]. When considering the potential efficacy of D-PDMP in a clinical setting, it should be noted that the compound is rapidly metabolized and cleared from the body. As such, another report by this same group shows that encapsulating the inhibitor inside of a biodegradable polymer composed of polyethylene glycol (PEG) and sebacic acid (SA) decreases its clearance rate by at least four orders of magnitude. Utilizing this method of delivery in mice maintains the positive effects on atherosclerosis progression described above, thereby demonstrating the potential therapeutic implications of D-PDMP treatment [236]. Indeed, this drug has also been shown to have beneficial effects on cardiac hypertrophy. When *Apoe^-/-^* mice on a high fat/high cholesterol diet are treated with D-PDMP, left ventricular mass, heart weight/body weight ratios, and myocardial fibrosis accumulation all decrease compared to placebo-treated animals. In addition, many echocardiographic functional parameters are improved, including fractional shortening and ejection fraction ratios. Preliminary data show that D-PDMP prevents cardiac hypertrophy by inhibiting MAPK phosphorylation [237]. These studies all suggest that inhibiting glycosphingolipid synthesis can have beneficial effects on atherosclerosis and cardiac hypertrophy, thereby leading to improved cardiac function. This may not be the only class of sphingolipids with deleterious effects on the heart. As an example, a recent report [238] utilized liquid chromatography-mass spectrometry of young and aged killifish, zebrafish, mouse, and human hearts to reveal a significant age-dependent accumulation of the lysosphingolipid sphinganine (dihydrosphingosine, DHS) across all species. Incubation of human pluripotent stem cell-derived cardiomyocytes (hCMs) with DHS led to DNA damage, as assessed by γ-H2A.X^+^ nuclear foci staining, which labels double-stranded breaks in the DNA [239]. In addition, DHS administration caused nuclear envelope disruption and increased histone acetylation, both signatures of increased aging [240,241]. Mechanistically, the authors showed that DHS derivatives inhibited histone deacetylase activity in hCMs, leading to the increased histone acetylation, and these effects were reversed by pharmacological inhibition of DHS converting enzymes and histone acetyltransferases. Interestingly, intraperitoneal administration of FB1 (a ceramide synthase inhibitor that increases endogenous DHS levels) to adult mice increased markers of aging in cardiomyocytes and also decreased fractional shortening and ejection fraction ratios in those mice. Inhibition of histone acetyltransferase activity with curcumin prevented these defects, thereby demonstrating that lysosphingolipids have a detrimental effect on cardiac aging and function both in vitro and in vivo [238].

## 4. Recent Advances in Bioactive Lipids in Cardiac Development

**Figure 3:**
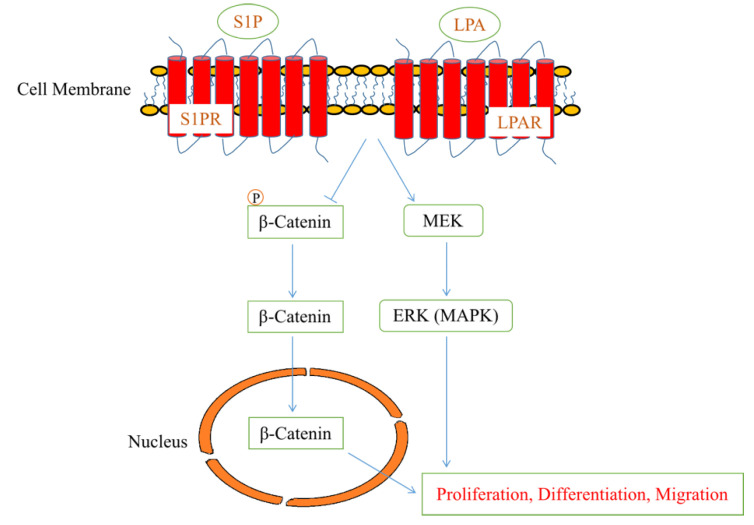
Lysophospholipid signaling in induced pluripotent stem cells (iPSCs). When introduced to iPSCs during the cardiomyocyte differentiation process, S1P and LPA bind to their respective GPCRs to activate the Wnt/β-catenin and MAPK signaling pathways. S1P and LPA facilitate nuclear localization of β-catenin, likely by increasing the available pool of its non-phosphorylated form. They also activate MEK (MAPKK), which then activates ERK (MAPK), leading to cell cycle progression. The downstream effects of S1P and LPA include enhanced cardiac differentiation of iPSCs and increased cellular proliferation and migration. The mechanisms described in this figure may recapitulate the pathways activated by lysophospholipids in the regulation of cardiovascular development in vivo. **Abbreviations:** GPCR, G-protein coupled receptor; iPSC, induced pluripotent stem cell; LPA, lysophosphatidic acid; LPAR, LPA receptor; MAPK, mitogen-activated protein kinase; S1P, sphingosine-1-phosphate; S1PR, S1P receptor.

There is ample evidence that lysophospholipids are vital in ensuring normal cardiac development, particularly sphingosine-1-phosphate. S1P specifically binds to S1P receptors to regulate novel functions in cardiac and lower jaw development in zebrafish [242]. It has already been demonstrated that the S1P receptor 2 controls the migration of heart cell precursors in the zebrafish embryo [243], but the effects of the S1P receptor 1 on development are more controversial. Several studies demonstrate that morpholino-mediated knockdown of the *s1pr1* gene results in excessive blood vessel sprouting and the formation of ectopic vessel branches, suggesting that this receptor functions to stabilize the developing vasculature in zebrafish [244,245]. However, transcription activator-like effector nuclease (TALEN)-mediated knockout of *s1pr1* expression does not result in any developmental defects, and these fish successfully grow into adults [246]. These apparent contradictions can be somewhat explained by a recent report that suggests that deleterious knockout mutations, but not morpholino-mediated knockdowns, activate compensatory responses in zebrafish vascular development [247]. In addition, some S1P receptors may have redundant functions in blood vessel formation. Morpholino-mediated knockdown of *s1pr1* alone causes only some degree of vascular defects, but knockdown of both *s1pr1* and *s1pr2* leads to severe vascular sprouting deficiencies [248]. These data suggest that after the loss of one S1P receptor isoform, other receptors may compensate to ensure that the vasculature develops normally. Notably, another recent study demonstrates that S1P receptor overexpression may have detrimental effects on zebrafish. *s1pr1* levels are normally suppressed by microRNA (miR)-19a, and when levels of this microRNA are decreased, higher *s1pr1* expression causes impaired cardiac looping, abnormal chamber shapes, and downregulation of cardiac precursor genes. *s1pr1* upregulation appears to mediate the detrimental effects on cardiac and fin development normally caused by T-box transcription factor 5 ((Tbx5) and subsequent miR-19a) depletion [249]. In mice, the situation appears to be much simpler, as shown by a recent report that utilized Cre-mediated conditional knockout of the S1P receptor 1 in developing embryos. This knockout caused ventricular noncompaction, ventricular septal defects, prevention of normal cardiomyocyte expansion, and perinatal lethality, demonstrating that *S1pr1* expression is necessary for normal cardiac development in mice [250]. It should also be noted that S1P has a developmental phenotype in vitro. A recent study utilized induced pluripotent stem cells (iPSCs) to show that S1P could enhance their differentiation to cardiomyocytes when administered at an early stage of the process and could increase CM proliferation when administered at a later stage. These effects are also replicated when the iPSCs are treated with lysophosphatidic acid, likely through modulation of canonical Wnt/β-catenin and ERK signaling pathways ([17]; see Figure 3 for a more detailed explanation of S1P and LPA effects in iPSCs). Indeed, LPA signaling has been implicated in zebrafish axis formation during body development and also controls proper heart looping and asymmetric expression of precursor genes in the heart [251,252].

Prostaglandins have long been known to play a role in cardiac development. One of the first pieces of evidence came in the 1970s when prostaglandin E_2_ (PGE_2_) was administered to four newborns with cyanotic congenital heart malformations in order to maintain arterial oxygen saturation levels until they were deemed ready for surgery to correct those defects [253]. It is now understood that prostaglandins maintain patency (openness) of the ductus arteriosus (DA), a blood vessel in the developing fetus that connects the pulmonary and systemic circulation. Prostaglandin levels are high during gestation to shunt blood into the systemic circulation, and they fall shortly after birth to close the DA and direct blood flow to the lungs [254]. A relatively recent study showed that these prostaglandin levels were controlled by the prostaglandin transporter (PGT). The authors utilized flippase (FLP) and Cre-mediated recombination to knockout PGT in mice, with none of the animals surviving past postnatal day 2. Upon post-mortem analysis, the knockout animals showed patency of the DA and abnormally dilated heart chambers. Administration of indomethacin, a non-selective cyclooxygenase inhibitor, to pregnant mothers led to lower PGE_2_ levels and normal closure of the DA in newborn pups, an effect that was attributed to PGT-mediated metabolism of prostaglandins [255]. PGE_2_ has also been shown to increase cellular communication network factor 3 (CCN3) mRNA and protein expression in the rat DA, leading to decreased intimal cushion formation. The intimal cushion is a smooth muscle layer that helps to close the DA, and these results further demonstrate the importance of prostaglandin signaling in controlling proper DA patency during development [256]. These effects are not just limited to one particular vessel. Microinjection of prostaglandin E_2_ into fertilized zebrafish eggs at the two-cell stage leads to increased embryonic tail flicks at 28 h post-fertilization (hpf), increased pigmentation at 48 hpf, and faster growth, hatching, and acquisition of movement by the embryos. In addition, at 96 hpf, the PGE_2_-treated larvae display increased blood vessel formation and maturity, as well as greater expression of vascular mRNA markers (*vegfa*, *kdrl*, *etv2*, *prox1*), compared to vehicle-treated animals. The authors of this report proposed that PGE_2_ activated PI3-kinase signaling, leading to increased Vegfa production, which then activated MAP kinase signaling and subsequent arterial and venous progenitor expansion [257]. In addition to in vivo experiments, some of the effects of prostaglandins can be modeled in vitro. In a recent study, Zhang et al. [258] utilized human ES cells to observe specification to the mesodermal lineage, which includes the heart. Upon BMP4 (bone morphogenetic protein 4) treatment, ES cells adopted a mesodermal fate and increased their secretion of PGE_2_. Forty-eight hours after indomethacin exposure to block endogenous PGE_2_ production, ES cells displayed decreased markers of mesodermal specification (*BRA*, *MIXL-1*, *EOMES*) and increased markers of neuroectodermal specification (*GBX2*, *HOXA1*, *ZIC1*). Interestingly, when exogenous PGE_2_ was added to the media, human ES cells committed to the mesodermal lineage (as assessed by qRT-PCR and immunostaining) even when BMP4 was not present. The authors demonstrated that PGE_2_ stimulated mesoderm differentiation by activating the prostaglandin EP2 receptor, which led to the stimulation of PKA activity, phosphorylation of GSK-3β, and subsequent activation of the canonical Wnt signaling pathway [258]. Clearly, prostaglandins utilize multiple mechanisms in order to regulate normal cardiovascular development.

Retinoids are another class of bioactive lipids that are known to play a role in cardiovascular development, as they are particularly important for proper formation of the cardiac outflow tract (OFT). Earlier studies demonstrate that retinoic acid (RA) deficient mice have shortened and misaligned OFTs [259]. In addition, the ectopic expression of RA downregulates *Tbx2* expression, leading to abnormal OFT development [260]. Both these reports suggest that altered RA levels affect normal transforming growth factor- β2 (TGF-β2) signaling, thereby causing the OFT defects. It is clear that retinoic acid must be expressed at the appropriate location and in the appropriate amounts for normal OFT development, and more recent reports have expanded upon these findings. Rydeen and Waxman [261] demonstrated that when the *cyp26a1* and *cyp26c1* genes were knocked down in zebrafish using morpholinos, RA was no longer degraded, leading to a decrease in ventricular cardiomyocytes in the first heart field. This decrease was due to the cardiomyocytes exiting the heart tube and undergoing apoptosis. Increased RA levels also prevented second heart field-derived progenitors from adding to the outflow tract, as they instead migrated to the developing pharyngeal arch arteries. The authors showed that excess RA decreased fibroblast growth factor 8a (*fgf8a*) expression and increased matrix metalloproteinase 9 (*mmp9*) expression. Notably, the introduction of a constitutively active Fgf receptor through heat shock restored the ability of second heart field cardiomyocytes to add to the OFT. In addition, the pharmacological inhibition of Mmp function rescued ventricular cardiomyocyte number and addition to the growing OFT, as well as the developing heart morphology, thereby suggesting a model where *fgf8a* and *mmp9* acted in parallel downstream of retinoic acid to maintain the integrity of the outflow tract [261]. This same group later demonstrated that Cyp26-deficient embryos displayed ectopic expression of *ripply3*, an important transcriptional co-repressor in zebrafish development. The histone deacetylase 1 (Hdac1) epigenetic regulator could repress *ripply3* transcription, thereby facilitating OFT development [262]. Importantly, the necessity of RA signaling has also been demonstrated in mouse embryos as the loss of retinaldehyde dehydrogenase 2 enzymatic activity decreases RA levels and leads to OFT defects and the development of persistent truncus arteriosus, a severe congenital heart defect [263]. More recent studies show the importance of retinoic acid for the development of parts of the outflow tract. For example, genetic mutation of the HECTD1 ubiquitin ligase (originally isolated by Kasarskis et al. [264]) decreases RA signaling in mice by influencing ubiquitination of its receptor and reducing activation of a receptor response element. These mouse embryos display numerous aortic arch abnormalities, including an aberrant right subclavian artery and a hypoplastic transverse arch, among others [265]. Still, other reports from the last several years show the importance of RA in development of the anterior lateral plate mesoderm [266], the compact zone of the ventricular myocardium [267], and the differentiation of smooth muscle cells relative to endothelial cells in coronary vessel walls [268]. The above studies combine to demonstrate that proper retinoic acid signaling is required for normal cardiovascular development.

## 5. Recent Advances in Bioactive Lipids in Cardiac Regeneration

MI is a leading cause of morbidity and often leads to immediate death due to lack of oxygenation in the ventricular muscle (usually by blockage of a coronary artery), which rapidly leads to ischemia/reperfusion injury and necrosis of the heart tissue [269,270]. In most cases, patients face a progressive decline of their condition over several years, finally resulting in heart failure. The lack of regenerative potential of the heart to restore the large numbers of lost cardiomyocytes leads to the formation of a non-contractile fibrotic scar that compromises heart function [271,272]. For all these reasons, there is significant interest in developing novel methodologies for replacing lost cardiac tissue. Until recently, cardiomyocytes have been considered fully post-mitotic cells, due in part to being multinucleated polyploid cells. Several findings have challenged this concept over the last 15 years and have demonstrated the ability of cardiomyocytes and the heart to endogenously regenerate; however, this response is insufficient for functional restoration [271,273,274]. As such, stem cell biology and cell reprogramming approaches hold great promise for ushering in a new era of cell-based therapy, sparking considerable interest among scientists, clinicians, and their patients [271,275,276,277,278]. Additionally, stem cell research can provide a greater comprehension of the endogenous and exogenous proteins, lipids, and signaling molecules that contribute to regeneration and repair for therapeutic purposes. The number of studies on bioactive lipids and cardiac regeneration has grown exponentially in recent years, and several have found a direct causal connection between the two [17,279]. Much of this work has been conducted using zebrafish, an excellent model for studying vertebrate regeneration. Zebrafish have the ability to efficiently regenerate injured cardiac cells and tissues as adults, a process that is limited at best in mammals [280,281]. This naturally occurring phenomenon can provide valuable information related to the potential reactivation of regeneration in mammalian studies.

Among bioactive lipids, vitamin D has recently been shown to promote regeneration in zebrafish. Han et al. [282] conducted a large-scale chemical screen in zebrafish utilizing the fluorescent ubiquitin-based cell cycle indicator (FUCCI) system [283] to detect proliferating cardiomyocytes in live embryos. They found that two vitamin D analogs—alfacalcidol (Alfa) and calcipotriene—both caused at least a doubling of proliferating CMs after 24 h of treatment. In addition, Alfa administration significantly increased animal growth and CM proliferation in adult zebrafish. Expression of a constitutively active form of the vitamin D receptor in cardiomyocytes also increased FUCCI^+^ CMs and led to cardiomegaly, and these trends were reversed with a dominant-negative form of the receptor. Importantly, alfa injection six days post ventricular resection resulted in a 41% increase in CM proliferation index compared to uninjured animals and knockdown of the vitamin D receptor blocked heart regeneration [282]. The authors utilized pharmacological inhibition of the ErbB2 receptor (a receptor tyrosine kinase, previously shown to promote cardiac regeneration in neonatal mice [284]) to conclude that vitamin D required this signaling pathway to exert its regenerative effects. Interestingly, recent reports also suggest that vitamin D promotes regeneration of the vasculature by inducing expression of stromal cell-derived factor 1 (SDF1; [285]). Supplementation of vitamin D_3_ in both humans and mice has increased the number of circulating angiogenic myeloid cells (AMCs), which are believed to promote vascular regeneration [286,287] and rescue impaired angiogenesis in diabetic mice. The authors found that vitamin D_3_ increased levels of hypoxia-inducible factor 1-α, which then promoted SDF1 expression. After the injury, SDF1 expression increased in mice and inhibition of C-X-C chemokine receptor type 4 (CXCR4), the SDF1 receptor, blocked homing of AMCs to the site of injury, and significantly reduced levels of vascular reendothelialization. Importantly, conditional knockout of the vitamin D receptor in myeloid cells also reversed its pro-regenerative effects, thereby proposing a mechanism by which vitamin D induced vascular regeneration after injury [285].

A major goal in cardiac research using human-induced pluripotent stem cells is to provide large quantities of cardiomyocytes suitable for cellular therapy in regenerative medicine. Human iPSCs can self-renew and differentiate into any cell type in the human body [288]. Therefore, they hold valuable promise in regenerative medicine as they offer the ability to develop in vitro models of regeneration and development and to obtain translational information for the treatment of human cardiac diseases. There are numerous stem and progenitor cell populations present in most tissues in the body, including the heart [289,290], and they can potentially be utilized to regenerate these tissues in conditions of disease or injury [291]. As an example, recent reports suggest that byproducts of oxylipins can influence stem cell processes. Specifically, Hsueh et al. [292] utilized cardiomyocyte-specific tamoxifen-inducible Cre-*LoxP* MerCreMer/ZEG (M/Z) mice [293,294] to observe the fate of endogenous stem/progenitor cells after injury. Ten days post-MI, ~20% of cardiomyocytes at the border zone were replaced by endogenous cardiac stem cells. Pre-treatment with exogenous prostaglandin E_2_ (PGE_2_) further increased CM replenishment by ~9%, and this restoration was prevented by pre-treatment with indomethacin (a cyclooxygenase pathway inhibitor that blocks prostaglandin synthesis). Interestingly, intramyocardial injection of cardiac progenitor cells after injury led to increased cardiomyocyte differentiation, but only if they expressed the prostaglandin EP2 receptor. Finally, Hsueh and colleagues demonstrated that PGE_2_ administration also facilitated stem-cell mediated CM replenishment after injury in aged mice by inhibiting the expression of *Tgf-β1* [292]. Another recent study suggests that PGE_2_ can interfere with the ability of fibroblasts to reprogram into cardiomyocyte-like cells by acting through the EP4 receptor. Here, Muraoka et al. [295] utilized a high-content screening of 8400 chemical compounds to identify an anti-inflammatory factor (diclofenac) that drove cardiac reprogramming of mouse adult fibroblasts to CMs in the presence of *Gata4*, *Mef2c*, and *Tbx5*. Notably, the addition of an EP4 receptor antagonist further enhanced reprogramming, an effect that was replicated by the siRNA-mediated knockdown of EP4 (*Ptger4*) in mouse tail-tip fibroblasts. As expected, an EP4 agonist completely blocked cardiac reprogramming. The authors concluded that diclofenac enhanced fibroblast reprogramming ability by blocking PGE_2_/EP4 signaling as well as the subsequent activation of cyclic adenosine monophosphate (cAMP)/protein kinase A and interleukin-1β pathways [295]. The above results illustrate that different prostaglandin signaling pathways can have differential effects on cardiac stem cells and reprogramming in the context of regeneration.

Retinoids are also known to play a role in cardiac regeneration. When the retinaldehyde dehydrogenase enzyme is knocked out, fetal mouse hearts display an increased number of cardiac progenitor cells, but these cells are unable to differentiate into cardiomyocytes, likely due to a decrease in fibroblast growth factor (FGF) signaling [180]. A particularly noteworthy study has shown that retinoic acid produced by the endocardial and epicardial layers of the heart is essential for its regeneration after injury. One day after ventricular resection surgery, zebrafish increase *raldh2* expression in these regions, leading to increased RA levels. Transgenic inhibition of RA or the use of RA-degrading enzymes prevents cardiomyocyte regeneration, and these results are also reproducible in other fish models [296]. More recent reports have expanded upon these findings. Drowley et al. [297] utilized a hiPSC-derived cardiac progenitor cell (CPC) assay, described previously [298], to screen 10,000 different compounds for their proliferative effects. They found that two agonists of the retinoic acid receptor (RAR)—ATRA (all-trans retinoic acid) and AM580—increased the number of CPC nuclei without altering their inherent progenitor cell identity, an effect that was attenuated by RAR antagonists. When applied to CPCs on day 5 of the cardiac differentiation protocol, RA inhibited terminal cardiomyocyte differentiation and activated human CPC proliferation instead, demonstrating the stage-specificity of this effect [297]. Another recent study utilized an inhibitor of retinoic acid synthesis in developing mouse embryos to show that a deficiency in RA signaling delayed the migration of embryonic epicardial cells into the myocardium, with the opposite effect being seen under conditions of excess RA production. The authors attributed these effects to the Ras homolog family member A (RhoA) signaling pathway [299]. This finding is notable because, during embryonic development, epicardial cells undergo an epithelial-to-mesenchymal transition to form epicardium-derived progenitor cells (EPDCs), which serve as multipotent precursors of cardiomyocytes and other cardiac cell types. This transition also occurs to an extent after cardiac injury, although the epicardium mediated regenerative response is insufficient to completely restore lost cardiomyocytes and cardiac function [300,301,302]. The above report suggests that inhibiting RA signaling in the epicardium (the main source of RA in the embryonic heart) may prevent the necessary cytoskeletal rearrangements that are necessary for EPDC infiltration of the myocardium in development and after injury, thus implicating retinoids as crucial mediators of cardiac regeneration [299].

Adult cardiomyocytes in mammals and lower vertebrates have a significant variation in proliferative capacity, likely due to phylogenetic and/or ontogenetic factors. Translating these factors could be helpful for the development of new alternative strategies that promote cardiomyocyte proliferation [303]. In the past, various molecular pathways have been investigated for their ability to cause cardiomyocyte proliferation both in mammals and fish: Wnt/β-catenin, TGFβ-activin, CDK9/PTEFb, insulin-like growth factor (IGF), reactive oxygen species (ROS), Hippo/Yes-associated protein (YAP)/transcriptional co-activator with PDZ-binding motif (TAZ), Meis1, miRNAs, hypoxia, and macrophages [281]. The Hippo/Yap/Taz pathway appears to be crucial in improving cardiac regeneration, as this pathway has an important role both in heart development and in postnatal cardiomyocyte proliferation [304,305]. It is notable that bioactive lipid signaling molecules are capable of regulating pluripotency and cell cycle activity in human pluripotent stem cells by stimulating and interacting with essential cellular signaling pathways, such as the Wnt/β-catenin pathway, the MAPK/ERK pathway, and the Hippo pathway [305,306]. In addition, stem cell differentiation can be modulated by pharmacologic compounds, including inhibitors of enzymes in lipid metabolism, lipid analogs, and drugs targeting downstream effectors of lipid-mediated signaling pathways, and, as such, these may be promising drug targets that can be used for in vitro differentiation of stem cells and eventual regenerative therapies [10,307].

**Figure 4:**
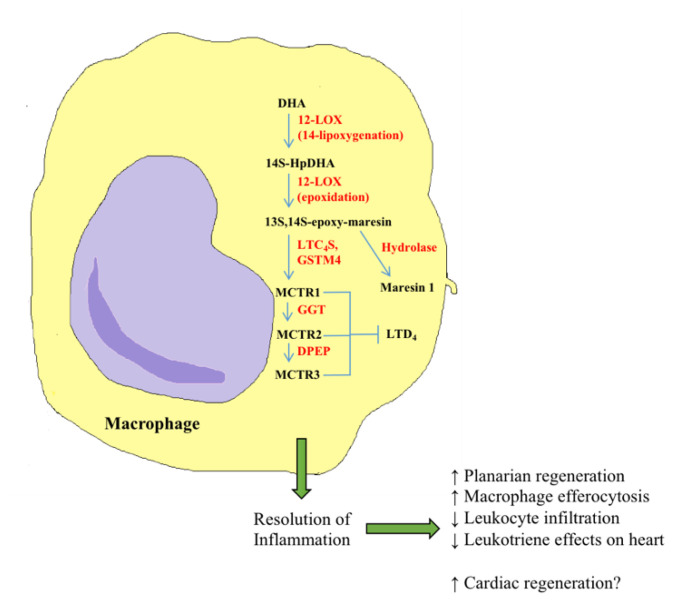
Maresin biosynthetic pathways in the resolution of inflammation. In human macrophages, DHA is converted to a 13,14-epoxide intermediate through the actions of LOX enzymes. This intermediate can then be hydrolyzed into maresin 1 or conjugated to peptides at position 13 on the DHA backbone to form MCTRs. The bioactive maresins and MCTRs produced by macrophages play a role in the resolution of inflammation, partly through countering the pro-inflammatory effects of leukotrienes, and this activity may be key to facilitating cardiac regeneration. **Abbreviations:** DHA, docosahexaenoic acid; DPEP, dipeptidase; GGT, γ-glutamyl transferase; GSTM4, glutathione S-transferase µ4; HpDHA, hydroperoxy-docosahexaenoic acid; LOX, lipoxygenase; LTC_4_S, leukotriene C_4_ synthase; LTD_4_, leukotriene D_4_; MaR1, maresin 1; MCTR1/2/3, maresin conjugate in tissue regeneration 1/2/3.

It should also be noted that the inflammatory response can affect regenerative outcomes, and several bioactive lipids have been implicated in these pathways. When uncontrolled and unresolved, acute inflammation can lead to tissue damage and chronic inflammation. In general, specialized pro-resolving mediators (SPMs, described above) facilitate the active resolution of this inflammation, and recent evidence suggests that this process may play a role in tissue regeneration [308]. One particular class of SPMs that has garnered extensive interest in regeneration is the maresins (macrophage mediators in resolving inflammation), which are produced by macrophages via 14-lipoxygenation of DHA [309]. In 2012, Serhan and colleagues discovered that maresin 1 (MaR1) accelerated the rate of regeneration in planarian worms after post pharyngeal head resection compared to controls and to other SPMs. The worms also biosynthesized MaR1 after injury, possibly to stimulate tissue regeneration [310]. These findings were further expanded when Dalli et al. [311] discovered two compounds with 14-hydroxylated DHA conjugated to peptides at position 13 on the DHA backbone, later termed maresin conjugates in tissue regeneration (MCTRs). These compounds were initially found in mouse exudates following self-resolving *Escherichia coli* infection and in human milk, and when introduced to injured planarian worms, decreased the time to 50 percent regeneration by about one day. The exudates were shown to facilitate regeneration by the upregulation of ERK signaling. Their chemical compositions were determined by liquid chromatography-tandem mass spectrometry (LC-MS-MS), and when the purified MCTRs were administered to planarian worms after *E. coli* infection, similar pro-regenerative phenotypes were observed. Notably, MCTR1 and 2 resolved *E. coli* infections in mice, as evidenced by increased bacterial phagocytosis. In addition, they also prevented reperfusion injury in mouse lungs by decreasing leukocyte infiltration and upregulating Ki67 and R-spondin protein levels [311]. Later studies identified a third MCTR, which also promotes planarian regeneration and resolves *E. coli* infections in mice [312]. Interestingly, MCTR biosynthesis and activity seems to be related to that of leukotrienes. In human macrophages, MCTR1 is derived from DHA via an epoxide intermediate through the actions of leukotriene C_4_ synthase, and inhibition of this enzyme reduces MCTR levels but increases the levels of other SPMs, such as resolvins and epoxins. The gamma-glutamyl transferase (GGT) enzyme converts MCTR1 to MCTR2, which is then hydrolyzed to MCTR3 by dipeptidase enzymes. These enzymes are also shared with leukotriene metabolic pathways [313]. It is perhaps somewhat surprising that MCTR1 and 2 significantly reduce leukotriene-mediated vascular leakage in mice and block the negative chronotropic effects induced by leukotrienes in sea squirt hearts. It appears that MCTRs directly interact with cysteinyl leukotriene receptor-1, possibly antagonizing the effects of leukotriene D_4_ in the cardiovascular system ([314]; see Figure 4 for an overview of the pro-resolving maresin biosynthetic pathways that may be involved in cardiac regeneration). Clearly, the inflammatory interactions that regulate cardiac function are complicated, and more work is necessary in order to truly elucidate the effects of maresins and other SPMs in regeneration. Refer to Table 2 for a more detailed list of oxylipin functions related to inflammation and cardiovascular regeneration.

## 6. Conclusions

Recent advances in lipidomics, mass spectrometry, and lipid biology in the last few decades reveal that bioactive lipids harbor many biological functions outside of those traditionally associated with immunity and inflammation, including the control of cellular signaling pathways in cardiovascular disease, development, and regeneration [329,330]. These experimentally proven in vivo and in vitro findings are broadening our understanding of cardiovascular biology and will help to develop novel therapeutic strategies for CVD. Future genetic and pharmacological approaches to elucidate and better characterize these lipid signaling pathways will be necessary before their translation into the clinical setting.

## Figures and Tables

**Table 1:** Specific oxylipins and their functions in cardiovascular disease.

Oxylipins in Cardiovascular Disease		
Lipid	Beneficial Function	Reference
11,12-EET	Suppresses cardiac hypertrophy and increases ANP levels in mouse hearts Prevents high-fat diet-induced atherosclerosis in mice	[200] [201]
14,15-EET	Decreases ventricular pressure and reduces pulmonary artery wall thickness in rats	[204]
CYP2J-Derived EETs	Improves left ventricular function and reduces collagen accumulation after MI in mice	[193]
sEH Inhibitors	Prevent ischemia-reperfusion injury in rat heart isolates	[203]
19-HETE	Ameliorates angiotensin II-induced cardiac hypertrophy in rats Reduces platelet aggregation in mouse blood vessels	[196] [198]
15-epi-lipoxin A_4_	Improves ejection fraction and facilitates neutrophil clearance after coronary artery ligation in mice Reduces intimal hyperplasia after carotid artery ligation in mice	[192] [207]
ATL A4	Prevents atherosclerotic lesions in rat aorta by resolving inflammation	[206]
EPA/DHA	Lower serum triglyceride levels in patients Reduce adverse ventricular remodeling and myocardial fibrosis in patients Reduce the incidence of ischemia, severe cardiac events, and cardiovascular death in patients Decrease fibrosis accumulation after MI in rat hearts	[185] [186] [187] [189]
RvD_n-3 DPA_	Decreases leukocyte and platelet activation in patient peripheral blood samples	[188]
RvD1	Improves left ventricular function, promotes resolution of inflammation, and reduces collagen deposition after MI in mice Decreases oxidative stress, fibrosis, and necrosis and promotes the stability of atherosclerotic plaques in mice	[190] [194]
RvD4	Improves thrombus resolution in mice	[195]
PD1	Reduces inflammatory cell infiltration and neointimal hyperplasia after carotid artery injury in rats	[209]
MaR1	Reduces necrosis and promotes the stability of atherosclerotic plaques in mice	[210]
**Lipid**	**Detrimental Function**	**Reference**
16-HETrE	Increases blood pressure in patients	[211]
5,6-diHETrE	Increases blood pressure in patients	[211]
TXB_2_	Increases blood pressure in patients	[211]
LTB_4_	Antagonism reduces infarct size and inflammatory cell accumulation after coronary artery ligation in mice Inhibition increases the stability of atherosclerotic plaques in mice	[214] [217]
LTC_4_	Receptor inhibition increases ejection fraction and myocardial mass after cardiac cryoinjury in mice	[215]
**Abbreviations**: ANP, atrial natriuretic peptide; ATL, aspirin-triggered lipoxin; CYP, cytochrome P450 enzyme; DHA, docosahexaenoic acid; diHETrE, dihydroxyeicosatrienoic acid; EET, epoxyeicosatrienoic acid; EPA, eicosapentaenoic acid; HETE, hydroxyeicosatetraenoic acid; HETrE, hydroxyeicosatrienoic acid; LT, leukotriene; MaR, maresin; MI, myocardial infarction; PD, protectin; RV, resolvin; sEH, soluble epoxide hydrolase; TX, thromboxane		

**Table 2:** Specific oxylipins and their functions in cardiovascular regeneration.

Oxylipins in Cardiovascular Regeneration		
Lipid	Function	Reference
5,6-EET 8,9-EET	Stimulate proliferation in murine microvascular endothelial cells and angiogenesis in mice	[315]
8,9-EET	Attenuates apoptosis in primary rat cardiac myocytes after hypoxia and reoxygenation	[316]
11-HETE	Inhibits proliferation of human vascular smooth muscle cells	[317]
15-HETE	Inhibits PMN migration across cytokine-activated human endothelium cells in culture	[318]
20-HETE	Stimulates proliferation of rat aorta vascular smooth muscle cells Stimulates inflammatory cytokine production in human vascular endothelial cells	[319] [320]
5-oxo-ETE	Stimulates human eosinophil migration	[321]
15-oxo-ETE	Inhibits human vascular vein endothelial cell proliferation	[322]
18-HEPE	Inhibits macrophage-mediated activation of murine cardiac fibroblasts and prevents pressure overload-induced cardiac fibrosis and inflammation in mice	[323]
14S,21-diHDHA	Enhances wound healing in murine models	[324]
9,10-diHOME	Decreases left ventricular developed pressure and increases coronary resistance after ischemia/reperfusion injury in mice	[325]
HxA3	Recruits human PMN to sites of inflammation	[326]
LxA_4_ LxB_4_	Stimulate phospholipid remodeling without causing aggregation in human neutrophils	[327]
PGE_2_	Enhances cardiomyocyte replenishment after MI in young mice and rescues cardiomyocyte replenishment after MI in aged mice	[292]
	Inhibition enhances reprogramming of mouse tail-tip fibroblasts to cardiac cells	[295]
TxA2	Stimulates mitogenesis of guinea-pig coronary artery smooth muscle cells	[328]
MaR1	Accelerates planarian regeneration, increases human macrophage efferocytosis, decreases PMN infiltration in mice	[310]
MCTR1,2,3	Stimulate planarian tissue regeneration, increase macrophage efferocytosis, reduce neutrophil infiltration, counteract leukotriene effects on the heart	[311,312,314]
**Abbreviations**: diHDHA, dihydroxydocosahexaenoic acid; diHOME, dihydroxyoctadecenoic acid; EET, epoxyeicosatrienoic acid; HEPE, hydroxyeicosapentaenoic acid; HETE, hydroxyeicosatetraenoic acid; Hx, hepoxilin; Lx, lipoxin; MaR, maresin; MCTR, maresin conjugate in tissue regeneration; MI, myocardial infarction; oxo-ETE, oxo-eicosatetraenoic acid; PG, prostaglandin; PMN, polymorphonuclear leukocyte; Tx, thromboxane		

## Abbreviations

diHDHA	dihydroxydocosahexaenoic acid
diHOME	dihydroxyoctadecenoic acid
EET	epoxyeicosatrienoic acid
HEPE	hydroxyeicosapentaenoic acid
HETE	hydroxyeicosatetraenoic acid
Hx	hepoxilin
Lx	lipoxin
MaR	maresin
MCTR	maresin conjugate in tissue regeneration
oxo-ETE	oxo-eicosatetraenoic acid
PG	prostaglandin
PMN	polymorphonuclear leukocyte
Tx	thromboxane
